# ESCO2 promotes hypopharyngeal carcinoma progression in a STAT1-dependent manner

**DOI:** 10.1186/s12885-023-11527-5

**Published:** 2023-11-15

**Authors:** Juan Hu, Jing Yan, Yijie Chen, Xiaohui Li, Liu Yang, Haiyu Di, Huihui Zhang, Yewen Shi, Junjie Zhao, Yanxia Shi, Yinglong Xu, Xiaoyong Ren, Zhenghui Wang

**Affiliations:** 1 Department of Otorhinolaryngology, Head and Neck Surgery, the Second Affiliated Hospital of Xi’an Jiaotong University, Xi’an, Shaanxi China; 2 Department of Maxillofacial Surgery, Affiliated Stomatological Hospital of Xi’an Jiaotong University, Xi’an, Shaanxi China

**Keywords:** ESCO2, HNSC, Hypopharyngeal carcinoma, Malignant progression, STAT1

## Abstract

**Background:**

The establishment of sister chromatid cohesion N-acetyltransferase 2 (ESCO2) is involved in the development of multiple malignancies. However, its role in hypopharyngeal carcinoma (HPC) progression remains uncharacterized.

**Methods:**

This study employed bioinformatics to determine the ESCO2 expression in head and neck squamous cell carcinoma (HNSC) and normal tissues. In vitro cell proliferation, migration, apoptosis, and/or cell cycle distribution assays were used to determine the function of ESCO2 and its relationship with STAT1. Xenograft models were established in nude mice to determine ESCO2 in HPC growth in vivo. Co-immunoprecipitation/mass spectrometry (Co-IP/MS) was conducted to identify the potential ESCO2 binding partners.

**Results:**

We found that ESCO2 expression was elevated in HNSC tissues, and ESCO2 depletion suppressed tumor cell migration in vitro and inhibited tumor growth in vitro and *in vivo.* Co-IP/MS and immunoblotting assays revealed the interaction between ESCO2 and STAT1 in HPC cells. STAT1-overexpression compromised ESCO2-mediated suppressive effects on HPC cell proliferation, viability, and migration.

**Conclusions:**

These findings suggest that ESCO2 is crucial in promoting HPC malignant progression through the STAT1 pathway and provides novel therapeutic targets for HPC treatment.

**Supplementary Information:**

The online version contains supplementary material available at 10.1186/s12885-023-11527-5.

## Background

Hypopharyngeal carcinoma (HPC) originates from the hypopharyngeal mucosal epithelium, occupying 5% of head and neck malignancies. The overall 5-year survival rate is lower than 20%, indicating that the therapeutic strategies are not favorable [1]. Therefore, there is an urgent need to find novel HPC therapeutic targets.

The establishment of sister chromatid cohesion N-acetyltransferase 2 (ESCO2) is a well-documented critical molecule during the cell cycle progression [2]. Importantly, ESCO2 depletion significantly inhibited cell proliferation and promoted cellular apoptosis [3]. Additionally, accumulating studies have associated ESCO2 with tumorigenesis. Elevated ESCO2 has been shown to promote tumor progression *via* leveraging different pathways, including p53, mTOR, and hnRNPA1 [3–6]. However, how ESCO2 facilitates HPC progression and the underlying mechanism are poorly defined.

STAT1, upon phosphorylation, can dimerize and enter the nucleus to promote gene transcription [7, 8]. Moreover, although the significance of STAT1 in tumor biology has been studied for a decade, the observations are still controversial [9]. Even in the same cell type, the STAT1 functions differently depending on the genetic background [10]. Furthermore, there is a cross-control between STATs and mTOR, as the latter tends to phosphorylate STAT proteins directly or indirectly, which may determine the fate and function of various cell types [11].

On the other hand, ESCO2 was shown to regulate mTOR in tumor cells [3]; therefore, STAT1 may also be associated with ESCO2’s molecular activity. Herein, we speculate that ESCO2 may regulate head and neck squamous cell carcinoma (HNSC) progression in a STAT1-dependent manner. We analyzed ESCO2 gene expression in the RNA sequencing data of HNSC to test this hypothesis. We also studied how ESCO2 and STAT1 are involved in HNSC in vitro and *in vivo.* The findings will lead us to hold the key to improving HPC treatment.

## Methods

### Public datasets analysis

To interrogate the relationship between ESCO2 and the clinical-pathological characteristics of the HPC, the clinical data and RNA sequencing data from 44 normal specimens and 458 HNSC deposited in the TCGA database were downloaded from UCSC Xena Data Hubs (http://xena.ucsc.edu/). Forty-three samples had paired adjacent normal tissue and pathology information among the samples. The data were normalized (TMM [Mean of M-Values]), and ESCO2 expression was analyzed against different clinical stages, gender, and age. Clinical data of patients with different ESCO2 expression levels were retrieved, analyzed, and plotted with *tableone* package using R. Additionally, the R package “*survminer*” was used to determine the cut-off point of ESCO2 expression levels based on the maximally selected log-rank statistics. The influences of tumor stages and baseline variables on the outcome by Cox proportional hazards analysis. The baseline variables in the univariable analyses were gender and age.

### Cell culture

To evaluate the functional importance of ESCO2 in HPC pathogenesis in vitro, the FaDu cell line was acquired from the Cell Bank of Shanghai Institute of Biochemistry and Cell Biology, Chinese Academy of Sciences (RRID: CVCL_1218, stock number TCHu132). The cells were incubated at 37 °C in a humidified atmosphere with 5% CO_2_ and were cultured in Minimum Essential Medium (MEM) supplemented with 10% FBS.

### Construction of lentiviruses

The ESCO2-targeting or control shRNAs (shESCO2-1, shESCO2-2, shCtrl) were purchased from Genechem (Shanghai, China) (Table 1) and constructed into the GFP-expressing GV115 lentiviral vector (Genechem, China). For STAT1 overexpression, full-length STAT1 (NM_001384880) was built into the Ubi-MCS-SV40-Cherry-IRES-puromycin plasmid. Virus packaging and harvesting were conducted as previously described [12]. Harvested viruses were used to transduce FaDu cells (2 × 10^5^) seeded into a 6-well plate for further studies.

**Table 1:** The target sequences of the ESCO2-specific shRNA oligos

Number	Sequence
shESCO2-1	AACACCAGATGGCAAGTTA
shESCO2-2	ACCTTACTTGTTCTGAGAT

### Quantitative real-time PCR (qPCR) analysis

As previously reported, the relative expression of ESCO2 in cells was determined by qPCR [13]. In brief, RNA was isolated from FADU cells utilizing TRIzol solution (Pufei, China). Complementary DNA (cDNA) was synthesized using the ReverTra Ace™ qPCR RT Kit (Promega, USA) according to the manufacturer’s instructions. Gene amplification was conducted in Light Cycler 480 II Real-Time PCR System (Roche, Germany) using M-MLV reverse transcriptase (Progema, US). The primer sequences of ESCO2 and GAPDH are shown in Table 2. The ESCO2 expression level was quantified relative to GAPDH using the 2^−ΔΔCt^ method.

**Table 2:** The primer sequences used for qPCR in this study

Gene name	Sequence (5ʹ→3′)
GAPDH	F: 5’-TGACTTCAACAGCGACACCCA-3’ R: 5’-CACCCTGTTGCTGTAGCCAAA-3’
ESCO2	F: 5ʹ-ATCCCCAAGCTCTACGGAATG-3ʹ R: 5ʹ-CAAACAGCCAAACATGAAGCA-3ʹ

### Western blot analysis

Western blot analysis was performed as previously described [14]. In general, cells were solubilized in RIPA buffer (Fisher Scientific) containing a protease inhibitor cocktail (Sigma), and protein concentration was determined with the Pierce BCA Protein Assay Kit (Fisher Scientific). Subsequently, an equal amount of total cell lysate was separated in SDS-PAGE gels and transferred to polyvinylidene difluoride membranes (Millipore, USA). The membrane was blocked in 5% goat serum for 1 h and was incubated with primary antibodies at 4ºC overnight, followed by secondary antibodies (Table 3). The membranes were developed by chemiluminescent substrate, and the immunoreactive bands were visualized on X-ray films. The intensity of bands using the densitometric scanning module on the ImageJ software reflected the relative protein expression. GAPDH was used as the internal control.

**Table 3:** The antibodies used in this study

Antibody Name	Company	Catalog #
CACYBP	Sigma	HPA025753
CDH1	Cell Signaling Technology	3195s
CUL1	Abcam	ab75817
CTNNB1	Cell Signaling Technology	#8480
EIF2B1	Abcam	ab181186
ESCO2	Abcam	Ab107277
Flag	Sigma	F1804
GADPH	Santa Cruz	Sc-32,233
HDAC2	Abcam	ab32117
LGALS3	Abcam	ab76245
MATR3	Abcam	ab151714
STAT1	Abcam	ab3987

### Cell viability assay

To evaluate the effect of ESCO2 silencing on cell viability, FaDu cells (2 × 10^3^ cells/well) with or without ESCO2 depletion were seeded into 96-well plates and cultured for the indicated periods. Then, 20 µl of MTT solution (5 mg/ml, Genview) was added to each well and incubated for 4 h at 37 °C. After removing the liquid, insoluble formazan crystals were dissolved in 100 µl DMSO (Sigma, USA) in an orbital shaker. Subsequently, the absorbance was measured at 570 nm in a spectrophotometer (Tecan Infinite, Switzerland). The optical density was used to reflect the number of viable cells.

### Cell proliferation analysis

To determine how ESCO2 and STAT1 regulate cell proliferation, their expressions were modulated *via* lentivirus-based gene overexpression or knockdown techniques. Lentivirus packaging and cell transduction were carried out by Genechem Col, Ltd. (Shanghai, China). Specifically, lentiviral particles for ESCO2 knockdown (KD) and their corresponding negative controls (NC) express GFP; while those for STAT1 overexpression (OE) and the corresponding negative controls (NC) express RFP. Cells were divided into three groups based on the gene expression modifications: negative controls (NC + NC), ESCO2 knockdown (KD + NC), and ESCO2 knockdown in combination with STAT1 overexpressing (KD + OE). Subsequently, cells from each group were seeded into 96-well plates (2 × 10^3^/well) in triplicates. After that, the fluorescence intensity was determined by Celigo® Image Cytometer (Nexcelom) for 5 consecutive days to reflect cell proliferation [15].

### Wound healing assay

To evaluate if ESCO2 is involved in cell migration, GFP-positive FaDu cells (3 × 10^4^/well) with or without ESCO2 depletion were cultured in Oris^™^ cell migration plates with cell seeding stoppers in the incubator overnight. Then, the stoppers were removed, and the culture medium was replaced with the starving medium (1% FBS). Images were captured at indicated periods (0, 24, and 48 h) using an inverted fluorescence microscope. Cells observed in the wound area, the cell-free zone, were considered migrated cells. The wound closure rate was calculated using the formula: [(wound area at 0 h – wound area at 24 or 48 h)/ wound area at 0 h] × 100%.

### Transwell migration assay

As previously reported, cell migration was assayed using the Transwell system [16]. Transduced FaDu cells in 100 µl of FBS-free medium were loaded in the upper chamber, and 600 µl of normal culture medium was added in the bottom chamber. The Transwell system was then returned to the incubator for 48 h. Subsequently, the cells in the upper chamber were removed, and the migrated cells were fixed with 4% paraformaldehyde and stained with crystal violet. Migrated cells from each well were counted and photographed in 9 randomly selected fields.

### Cell cycle assay

Cell cycle assay was conducted to determine if ESCO2 is involved in cancer cell cycle progression. In brief, FaDu cells were collected and washed with ice-cold PBS twice. Then, the cells were fixed with 75% ice-cold ethanol overnight and resuspended in PBS. Subsequently, the cells were stained with podium iodide (PI) (Sigma, USA) and screened by a flow cytometer (Millipore, USA). The cell cycle phases were analyzed using ModFit software (ModFit, USA).

### Apoptosis analysis

According to the manufacturer’s instructions, cell Apoptosis was evaluated using an Annexin V-APC apoptosis detection kit (eBioscience, 88-8007). In brief, control or ESCO2-silenced cells were harvested, prepared into single-cell suspension in binding buffer, and incubated with annexin V-APC and PI at room temperature for 15 min in the dark. Subsequently, the cell apoptosis was determined by a flow cytometer (Millipore, USA), and the results were analyzed using FCS Express software (version 3.0; DeNovo).

### Human HPC xenografts in nude mice

To confirm the role of ESCO2 in HPC progression in vivo, a cell line-derived xenograft model was established in nude mice. GFP-positive control or ESCO2-silenced cells (shCtrl, shESCO2-1) subcutaneously inoculated in BALB/c nude mice (female, 4 weeks old) purchased from Lingchang Biotechnology (Shanghai, China). In brief, mice were randomly allocated to 2 groups (n = 10), and each mouse received 4.0 × 10^6^ transduced cells in 200 µl of PBS/Matrigel mixture (BD Biosciences) subcutaneously under the right armpit. After that, the mice underwent tumor volume measurement every three days, and tumor volumes were plotted against time. Tumor volume was calculated using the formula (L × W × W)×π/6, where L and W are the tumor length and width, respectively [17]. On day 20, the fluorescent signals of the tumors were collected by the IVIS Imaging System (Perkin Elmer, USA). After scanning, animals were sacrificed by an overdose of 2% pentobarbital sodium and confirmed dead by cervical dislocation. Then, tumors were harvested and weighed. Experiments were carried out according to the National Institute of Health Guide for Care and Use of Laboratory Animals and were approved by the Institutional Bioethics Committee of the Second Affiliated Hospital of Xi’an Jiaotong University.

### Co-immunoprecipitation/Shotgun Mass Spectrometry (Co-IP/MS) analysis

To identify the interactome of ESCO2 in HPC cells, FaDu stably expressing 3-Flag-tagged ESCO2 or vehicle vectors were solubilized in IP lysis buffer (ThermoFisher Scientific) supplemented with protease inhibitor cocktail and PMSF (Sigma) for 1 h at 4 °C. Cleared lysates (16,000 × g, 20 min) were processed for Co-IP using protein A beads conjugated with anti-Flag antibody (Invitrogen) at 4 °C overnight. The immobilized proteins were eluted with 3-Flag peptide (Sigma) and subjected to immunoblotting or Coomassie blue staining followed by proteomic analysis (shotgun mass spectrometry). Antibodies used for immunoblotting are listed in Table 3.

For MS, the separated protein in the Coomassie blue-stained gel was dehydrated, vacuum-dried, and digested as previously described [18]. The generated polypeptides were identified through liquid chromatography-tandem mass spectrometry on the Q-Exactive HF-X quadrupole-Orbitrap mass spectrometer (ThermoFisher Scientific). Protein identification was achieved in the MASCOT 2.6 database and analyzed by Proteome Discoverer 2.1 (ThermoFisher Scientific).

### Statistical analysis

Statistical analyses were performed with SPSS 19.0 (SPSS Inc., USA). Data are presented as means ± standard deviations (SD) of at least three independent experiments. Comparisons between the two groups were performed with the independent samples using the student’s *t*-test or Mann-Whitney non-parametric test. Multiple-group comparisons were performed by one-way analysis of variance with Bonferroni post-tests. The independence of categorical variables was analyzed using Fisher’s exact test. Asterisks were used to annotate statistical significance (*P < 0.05, **P < 0.01, ***P < 0.001).

## 3 Results

### ESCO2 was upregulated in HPC tissues and associated with HPC prognosis

**Fig. 1:**
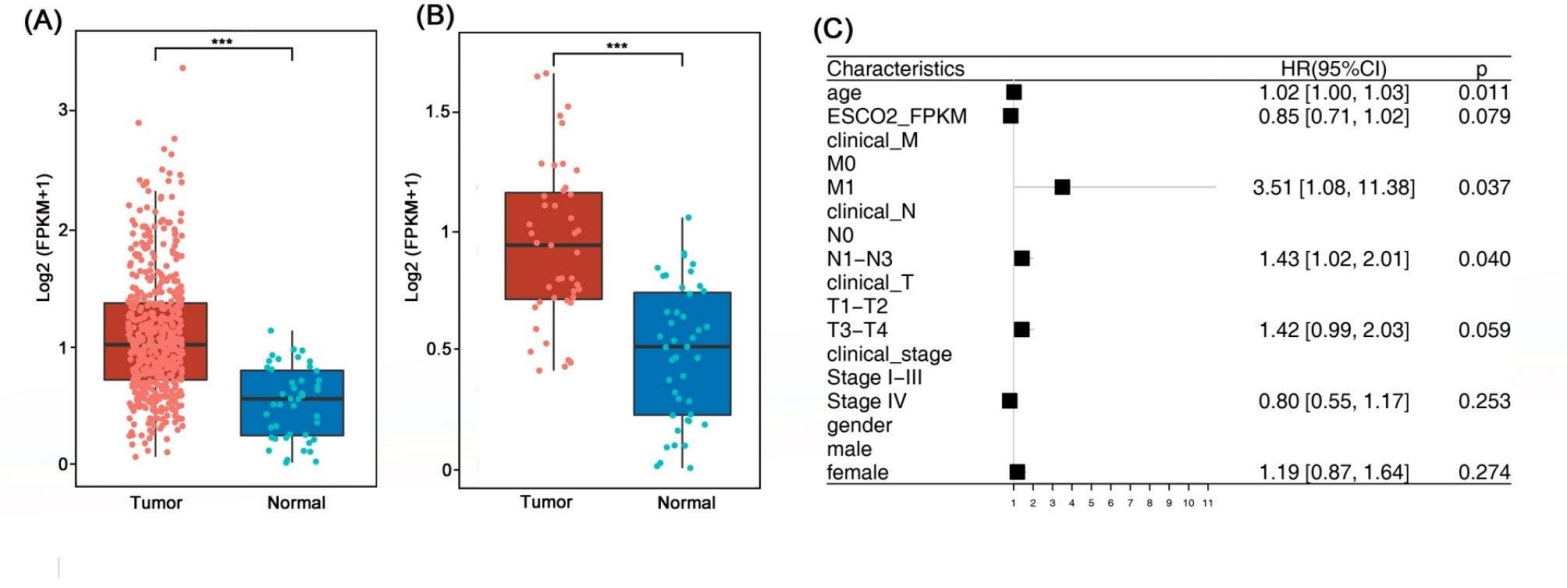
**ESCO2 expression in cancerous and normal tissue and HPC patients’ clinical features against ESCO2 expression. (A **and **B)** ESCO2 mRNA levels in HPC tissues and adjacent normal tissues from TCGA database (**A**) and patient samples (**B**). (**C**) A forest plot showing hazard ratios and 95% confidence intervals related to patient subgroups. Strata were age, ESCO2 expression level, TNM stages, clinical stages, and gender

First, we interrogated its expression level in tumor and normal tissues using a dataset from the TCGA database. The results showed elevated ESCO2 levels in HPC tissues compared with normal tissues (Fig. 1A, P < 0.001). Furthermore, the ESCO2 mRNA expression was compared in 43 paired specimens, and the results revealed significantly higher ESCO2 transcription levels in tumors than in paired-adjacent normal tissues (Fig. 1B, P < 0.001).

We then queried whether the ESCO2 expression level is involved in HPC patients’ tumor progression. We first allocated the patients into two groups based on their ESCO2 expression levels (ESCO2 high and ESCO2 low). Then, we retrieved the patients’ clinical information and checked whether the ESCO2 level was associated with the patients’ TNM stage, clinical stage, gender, and age (Table 4). Our data revealed that tumors with high ESCO2 expression are inclined to develop distant metastasis (M stage) (HR = 3.51, 95% CI = 1.08, 11.38, *P* = 0.037) and lymph node metastasis (N stage) (HR = 1.43, 95% CI = 1.01, 2.01, *P* = 0.04) (Fig. 1C). Together, these findings indicated that ESCO2 participates in the HNSC metastasis.

**Table 4:** Comparison of clinical features between HNSC patients with low and high ESCO2 levels in the TCGA database

Clinical characteristics	Clinical groups	ESCO2		*P*-value
		High (n = 223) (%)	Low (n = 223)(%)	
Clinical_M	M0	219 (98.2)	218 (97.8)	0.763
	M1	1 (0.4)	3 (1.3)	
Clinical_N*	N0	101 (45.3)	118 (52.9)	0.033
	N1-N3	121 (54.3)	99 (44.4)	
Clinical_T	T1-T2	87 (39.0)	74 (33.2)	0.2
	T3-T4	135 (60.5)	149 (66.8)	
Clinical_stage	Stage I-III	92 (41.3)	108 (48.4)	0.153
	Stage IV	131 (58.7)	115 (51.6)	
gender	female	53 (23.8)	65 (29.1)	0.238
	male	170 (76.2)	158 (70.9)	
age	<=60	116 (52.0)	103 (46.2)	0.256
	> 60	107 (48.0)	120 (53.8)	

### ESCO2 knockdown impaired HPC cell viability and proliferation

**Fig. 2:**
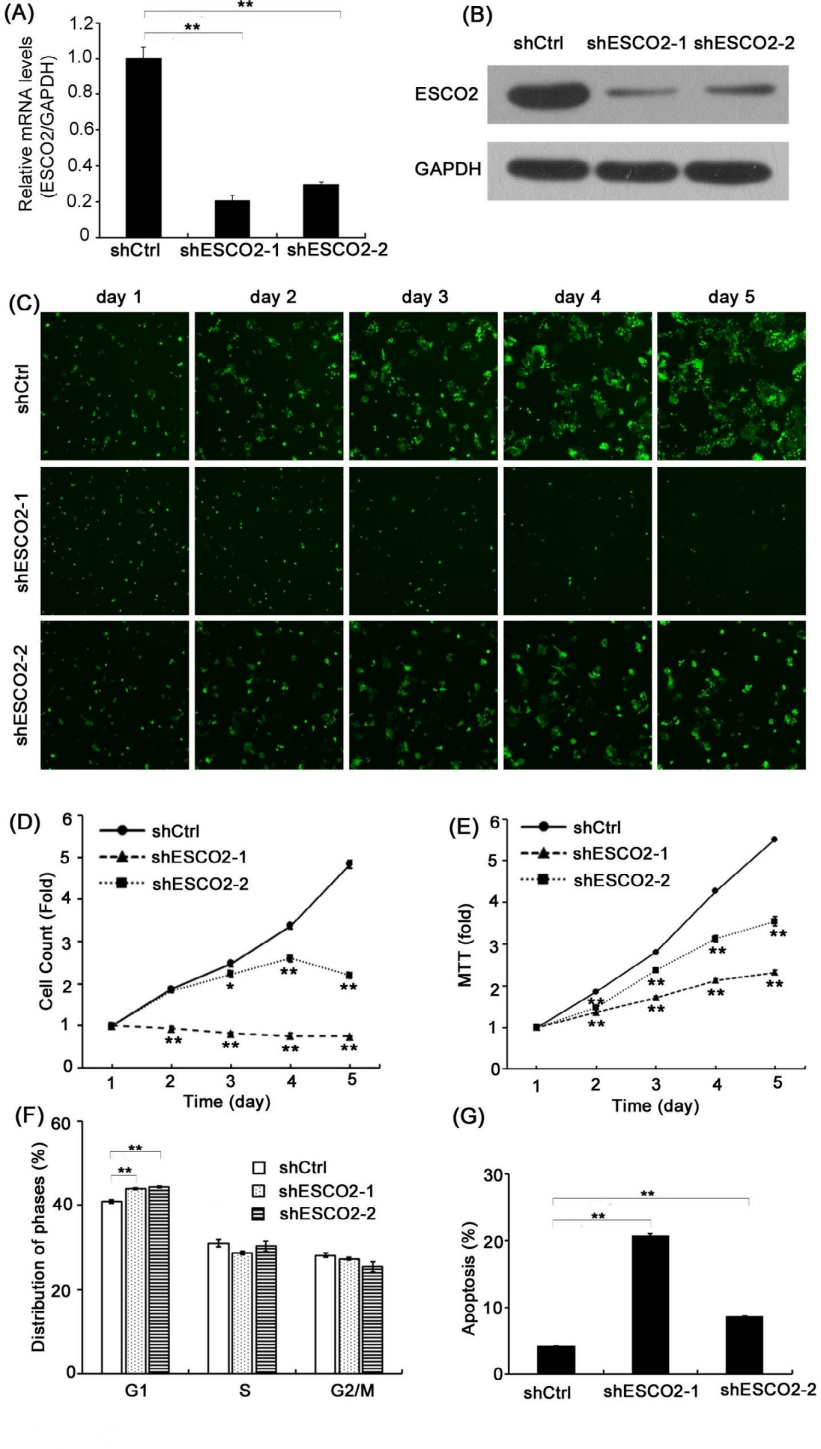
**Silencing ESCO2 inhibited HNSC cell growth and viability. (A **and** B)** The results of qPCR (**A**) and representative western blot analysis results (**B**) show shRNAs targeting ESCO2 (shESCO2-1 and shESCO2-2) sufficiently silenced in FaDu cells. **(C** and **D)** Representative images (**C**) and quantified data (**D**) of fluorescence-based proliferation assay showing ESCO2 deficiency inhibited HNSC cell proliferation at indicated time points (scale bar = 400 μm). (**E**) Analyzed results of MTT assay showing the ESCO2 depletion inhibited HNSC cell viability at indicated time points. (**F **and** G**) Analyzed results of the cell cycle analysis (**F**) and apoptosis assay (**G**), demonstrating ESCO2 knockdown affected HNSC cell cycle progression and apoptosis

To further explore the role of ESCO2 in HPC development, ESCO2 was silenced in FaDu cells by shRNAs (shESCO2-1 and shESCO2-2), and the ESCO2 knockdown was validated by qPCR and Western blotting analyses (Fig. 2A, B, P *<* 0.01). Subsequently, we interrogated the function of ESCO2 in cell proliferation; both shRNAs significantly suppressed the tumor cell growth since day 2 (Fig. 2C, D). Surprisingly, introducing shESCO2-1 completely abrogated the FaDu cell growth (Fig. 2C, D). In parallel, we evaluated the cell viability in those cells and found that HPC viability was significantly impaired by ESCO2 depletion (Fig. 2E). Moreover, the inhibitory effect on cell viability of ESCO2 depletion was observed as early as 2 days after the experiment’s initiation. Therefore, these data indicated that ESCO2 relates to the HPC proliferation and viability.

### ESCO2 knockdown elevated G1 phase population and induced cell apoptosis in HPC cells

Tumorigenesis is characterized by a disorganized cell cycle that leads to uncontrolled cellular proliferation [19]. Meanwhile, we observed that ESCO2 is closely related to cell growth and viability in HPC cells. Consequently, we sought to determine whether ESCO2 is involved in cell cycle progression. Thus, we compared the cell population in control or ESCO2-depleted cells at different cell cycle stages. Our results revealed that ESCO2 depletion led to a marginal increase in the G1 population (Fig. 2F, P < 0.01) but showed no significant difference in the S population.

Furthermore, we queried whether ESCO2 will affect apoptosis in HPC cells. The results demonstrated that ESCO2-targeting shRNA, especially ESCO2-1, significantly promoted cellular apoptosis in FaDu cells (Fig. 2G, P < 0.01). Specifically, in the control group, 4.23% of cells underwent apoptosis. Conversely, in cells expressing shESCO2-1 or shESCO2-2, the average apoptotic cell portion was 20.83% and 8.7%, respectively. Together, these findings suggested that ESCO2 appears to affect apoptosis in HPC cells significantly.

### ESCO2 promoted HPC cell migration

**Fig. 3:**
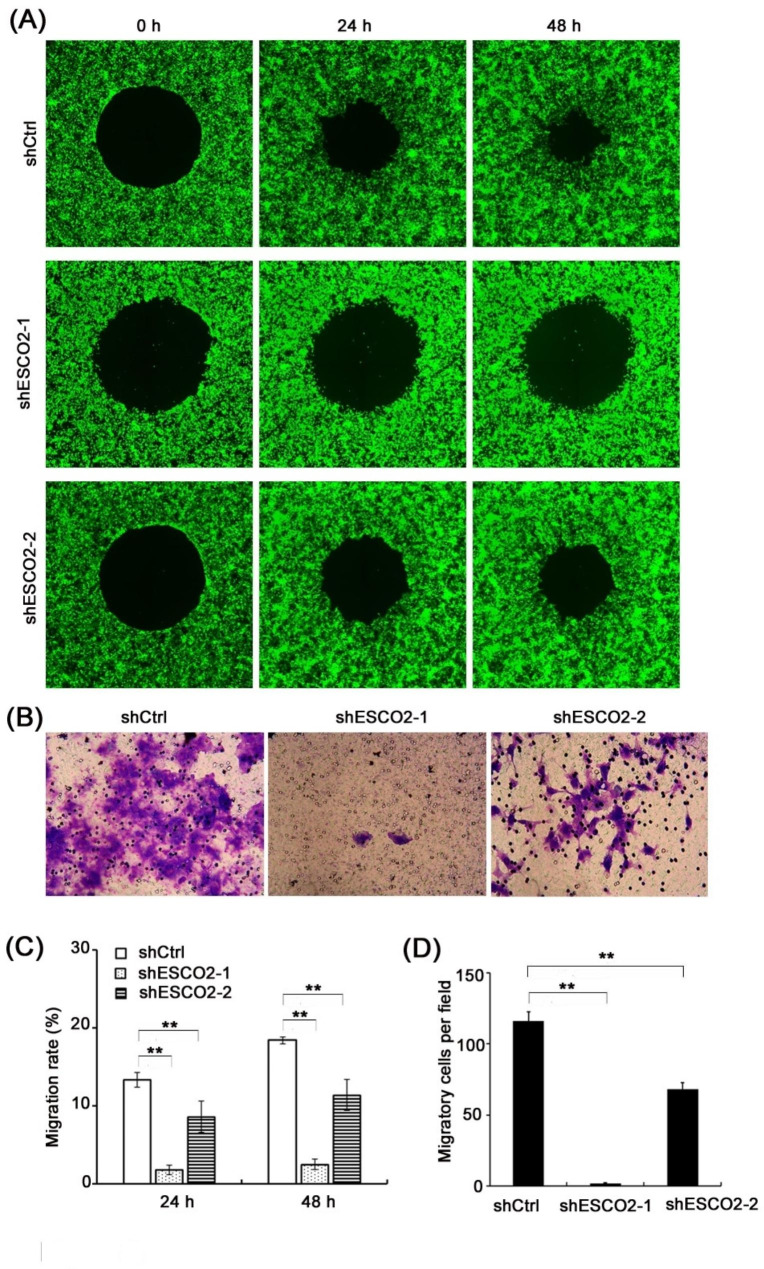
**ESCO2 depletion suppressed the HNSC cell migration. (A **and** C)** Representative images (**A**) and analyzed results (**C**) of fluorescence-based wound closure assay demonstrating the ESCO2 depletion inhibited FaDu cell migration. **(B **and** D)** Representative images (**B**) and statistical data (**D**) of the Transwell migration assay showing the effects of ESCO2 knockdown on FaDu cell migration potency (scale bar = 150 μm)

We then evaluated the importance of ESCO2 in cell migration. The wound-healing assay revealed that ESCO2 depletion significantly inhibited wound closure. As shown in Fig. 3A, the wound closing rates in the control group were approximately 50% and 75% at 24 and 48 h, respectively. However, the wound closure was significantly inhibited by ESCO2 depletion at the corresponding time points (Fig. 3A, C, P < 0.01). The Transwell migration assay was also conducted to confirm this result. We found that silencing ESCO2 inhibited the HPC cell migratory potential significantly compared with the cells transduced with control non-targeting shRNA (Fig. 3B, D, P < 0.01). More importantly, shESCO2-1 almost completely blocked the cancer cell migration (Fig. 3B, D, P < 0.01). Together, these data suggest that ESCO2 is involved in tumor cell migration.

### ESCO2-silencing restrained the HNSC progression in vivo

**Fig. 4:**
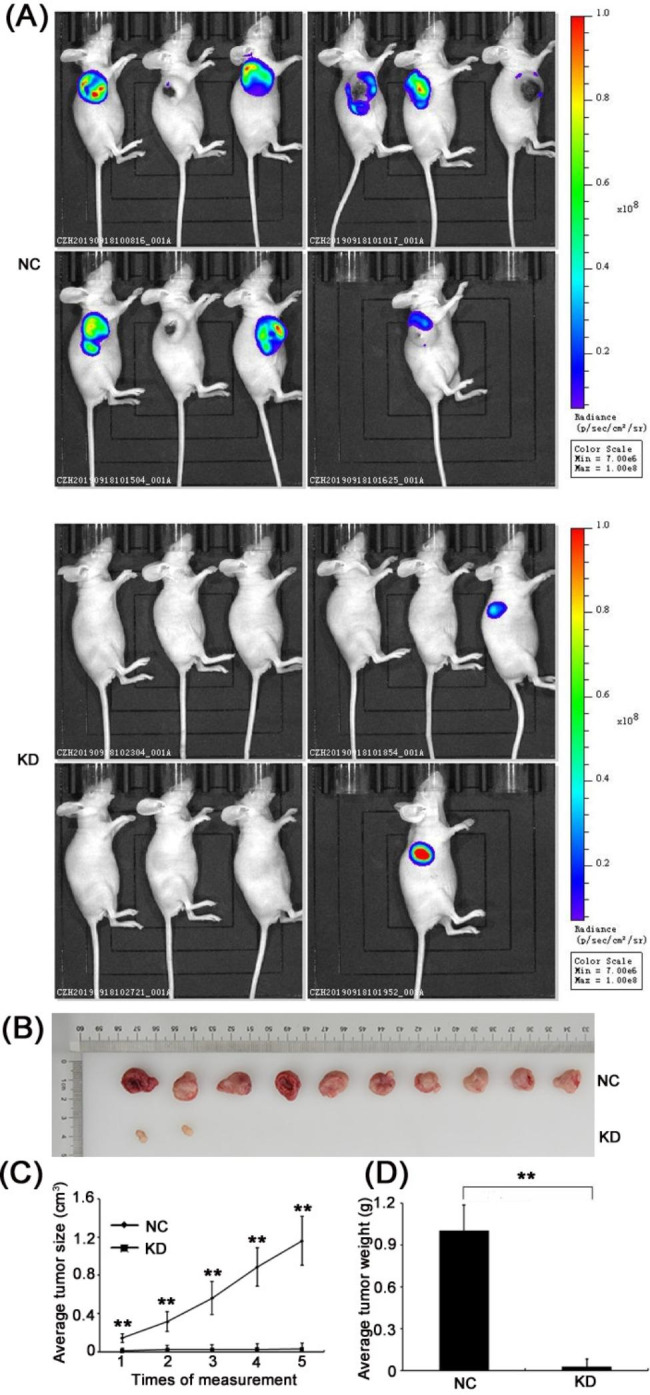
**Silencing ESCO2 restrained HNSC progression. (A** and** B)** Preclinical fluorescent (**A**) and photographic images (**B**) showing the tumor volumes of the control (NC) and ESCO2 deficient (KD) mice at the completion of the study. **(C)** A plot of the average tumor volumes for the mice from each group at indicated time points. (**D**) Analyzed results of average tumor weight for mice from each group

We established a subcutaneous xenograft in nude mice to understand further the functional importance of ESCO2 in HPC growth in vivo. FaDu cells transduced with lentivirus expressing GFP-ESCO2-targeting shRNA (KD) or GFP-control shRNA (NC) were injected subcutaneously into nude mice. Tumor volume was measured with a caliper and preclinical imaging system. As reflected by the marginal fluorescence signals, ESCO2-depleted FaDu cells barely grow into the detectable tumor in all tested mice (Fig. 4A upper panel, n = 10).

In addition, at the end of this study, we harvested the tumors from all mice and compared the volumes. Consistent with our preclinical imaging results, ESCO2-depleted cells hardly formed sizable tumors, while those transduced with control shRNA grew into large tumors (Fig. 4B-C, n = 10, P < 0.01). In parallel, the average tumor weight at the end of the study also revealed significant differences between these two groups (Fig. 4D, n = 10, *P* < 0.01). Together, these data suggest that ESCO2 is critical in regulating tumor growth in vivo.

### ESCO2 interacted with STAT1 in HPC cells

**Fig. 5:**
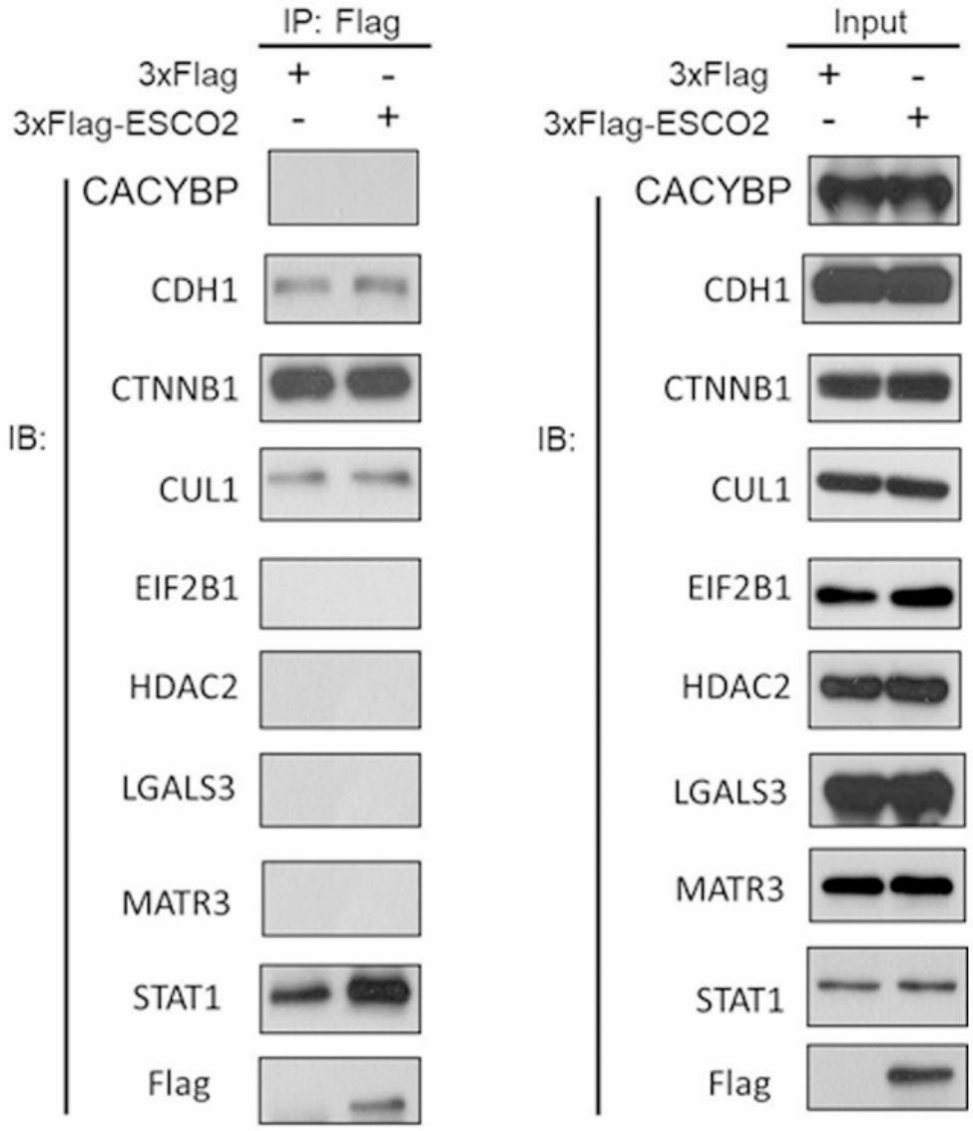
**ESCOt interacted with STAT1.** Representative images of Co-IP analysis evaluating the interactions between ESCO2 and Co-IP/MS-identified proteins that are closely related to cell migration and proliferation

Notably, the reconstitution of STAT1 attenuated the inhibitory effects on cell growth, viability, and migration achieved by ESCO2 silencing. Thus, we speculated that ESCO2 might contribute to the tumorigenesis of HPC by influencing STAT1. Furthermore, it has been demonstrated that ESCO2 can promote the methylation of histone H3 at lysine 9 (H3K9), which drives tumor growth in vivo [20] by forming macromolecular complexes [21]. Therefore, we speculated that ESCO2 might similarly promote HPC progression. As our data indicated that ESCO2-silenced cells showed significantly inhibited tumor cell growth, we sought to identify ESCO2’s interactome with a focus on cell migration and/or proliferation-related pathways. So, we performed Co-IP/MS analysis and identified the proteins that potentially interact with ESCO2. As a result, we identified 2349 peptides in the control group and 5590 peptides in the ESCO2 overexpressing group, which matched to 757 and 1270 proteins, respectively. From the candidates, we tested the interaction between ESCO2 and cell migration and/or proliferation-related proteins, including calcyclin-binding protein (CACYBP), cullin 1 (CUL1), cadherin 1 (CDH1), histone deacetylase 2 (HDAC2), catenin beta 1 (CTNNB1), martin 3 (MATR3), galectin 3 (LGALS3), and signal transducer and activator of transcription 1 (STAT1). The data indicated that ESCO2 interacts with STAT1 (Fig. 5). Notably, STAT1 depletion promotes HNSC progression and metastasis [22]. Therefore, it is reasonable to speculate that ESCO2 may interact with STAT1 to inhibit and impair its tumor suppressor activity. However, the molecular activity needs further investigation.

### STAT1-overexpression suppressed ESCO2-deficiency-mediated tumorigenesis

**Fig. 6:**
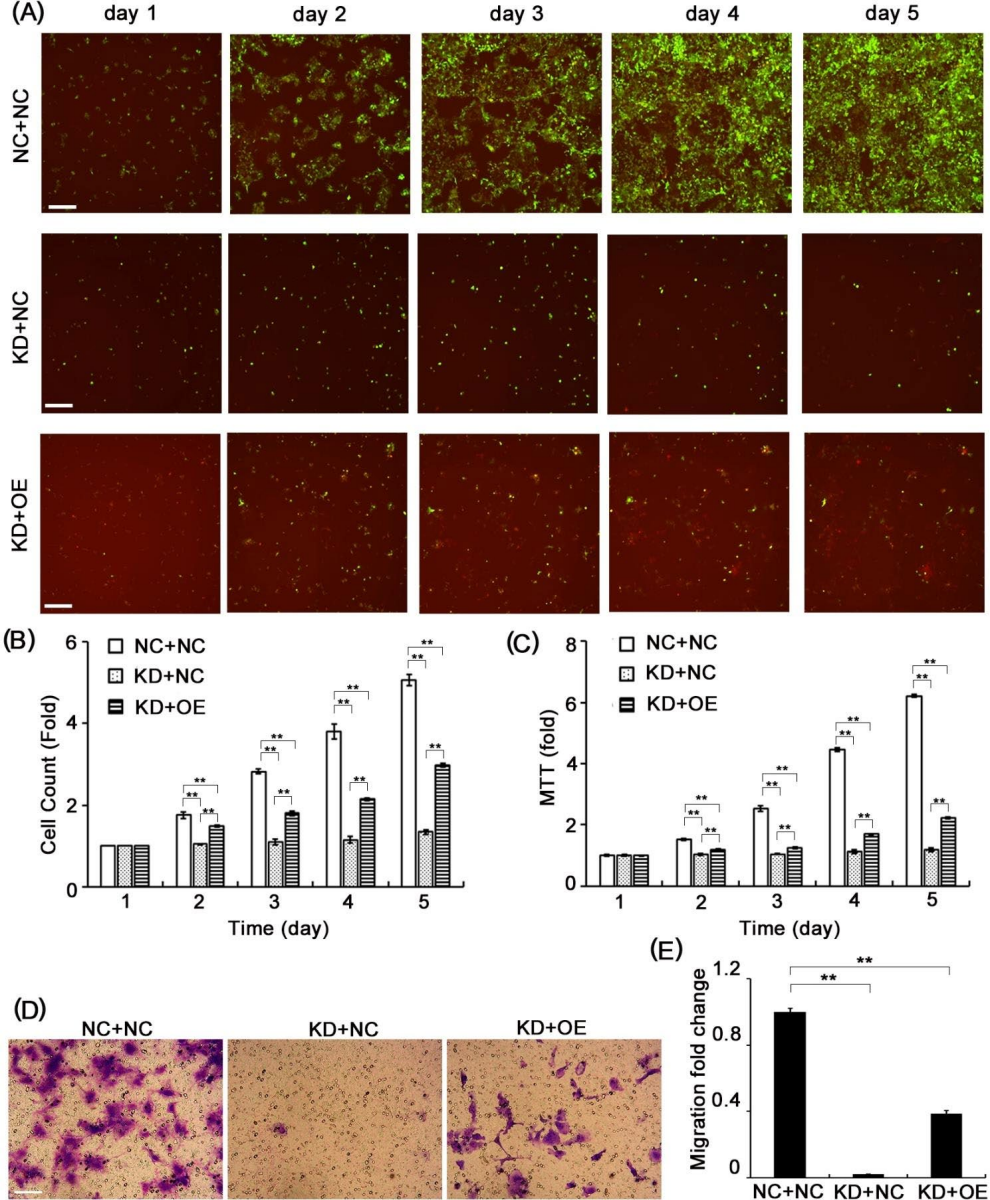
**Overexpression of STAT1 suppressed ESCO2 deficiency-mediated tumor progression** in vitro. **(A **and** B)** Representative images (**A**) and quantified data (**B**) of fluorescence-based proliferation assay evaluating the effects of STAT1 overexpression on cell proliferation in ESCO2-deficient cells (KD) at indicated time points (scale bar = 400 μm). Green fluorescence represents ESCO2 knockdown viral particles and the corresponding vehicle control; Red fluorescence indicates STAT1 overexpressing viral particles and the corresponding vehicle control. (**C**) Analyzed results of MTT assay detecting how STAT1 overexpression affects the cell viability in ESCO2-depleted cells at indicated time points. (**D **and** E**) Representative images (**D**) and analyzed results (**E**) of the Transwell migration assay showing STAT1 overexpression recovered the ESCO2 knockdown-mediated cell mobility inhibition in HNSC cells (scale bar = 150 μm). (NC + NC cells were transduced with control shRNA and control overexpression vehicles; KD + NC cells were transduced with ESCO2-targeting shRNA and control overexpression vehicles; KD + OE cells were transduced with ESCO2-targeting shRNA and STAT1-overexpressing viral particles.)

So far, our data have indicated that ESCO2 depletion significantly suppressed tumor growth in vitro and in vivo. In addition, a recent bioinformatics study demonstrated the potential significance of the STAT1/ESCO2 pathway in breast cancer [23]. It is reasonable to speculate that STAT1 may also be involved in ESCO2-associated tumorigenesis in HPC. Therefore, we investigated whether STAT1 plays a role in ESCO2-related HPC biology. Thus, we overexpressed STAT1 in ESCO2-depleted cells and evaluated how this modulation affects ESCO2-mediated cellular activities. First, we performed a proliferation assay and found that when ESCO2 was silenced, the cell proliferation was inhibited compared with that of the control group (Fig. 6A, B, P < 0.05).

Moreover, when we further overexpressed STAT1 in those proliferation-inhibited cells, the ESCO2 depletion-mediated cell growth suppression was significantly impaired (Fig. 6A, B, P < 0.05). Meanwhile, the cell viability assay also demonstrated that ESCO2 silencing significantly suppressed cell viability, but this effect was compromised by STAT1 overexpression (Fig. 6C, P < 0.05). We also evaluated whether STAT1 overexpression could counteract the ESCO2-depletion-mediated suppressive effect on HPC mobility. Consistent with our results, ESCO2-silenced cells exhibited reduced migratory potency compared with the control group. However, we also noticed that the ESCO2-deficiency-inhibited cell mobility was recovered when STAT1 was overexpressed (Fig. 6D, E, P *<* 0.05). Together, these results suggested a promotive role of STAT1 in HPC progression. More importantly, combined with observing the physical interaction between STAT1 and ESCO2, these findings indicated the involvement of STAT1 in ESCO2-mediated HPC progression.

## Discussion

Despite the improvements in surgery and drug development, the prognosis of patients with recurrent and metastatic HNSC who received traditional chemotherapy is less than 5% at one year [24]. Thus, it is critical to identify a more promising biomarker and therapeutic target for this malignancy.

This study found that ESCO2 was highly expressed in HNSC tumors compared with that in normal tissues, highlighting the importance of ESCO2 in HPC tumorigenesis. We also noticed that tumors expressing higher levels of ESCO2 are more prone to invade distant organs and lymph nodes. A recent investigation evaluated the ESCO2 expression in lung adenocarcinoma using multiple public databases, which echoes our study. The investigators found that tumors expressing high ESCO2 were associated with higher TNM stages [6]. These findings further compelled us to investigate the role of ESCO2 in HNSC development.

Moreover, our findings demonstrated that silencing ESCO2 restrained HPC’s growth and metastatic ability. These findings align with previous studies. For instance, downregulated ESCO2 gastric cancer cells promoted cellular apoptosis and suppressed tumor growth [3]. Also, elevated ESCO2 was connected to tumor progression and poor prognosis in renal cell carcinoma [5]. Also, lung cancer cells expressing high ESCO2 exhibited enhanced proliferative and metastatic potency [6]. All these data suggest ESCO2 plays a crucial role in the proliferation and metastasis of various human cancers. However, some researchers also reported a tumor-suppressive role of ESCO2 [25]. For instance, the author found colorectal cancer patients with higher ESCO2 expression had a longer overall survival rate, and ESCO2-depletion enhanced colon cancer cell migration. This inconsistency may be attributed to organ specificity.

Moreover, our study shows that ESCO2 is closely related to HPC cell migration and proliferation. Meanwhile, ESCO2 was reported to regulate tumor progression *by* forming a macromolecular complex to interfere with signaling transductions [6, 26]. Therefore, ESCO2 may exert regulatory effects on those cellular activities by interfering with corresponding signaling pathways in a similar manner. Thus, we investigated ESCO2 interactome focusing on cell migration and proliferation-related pathways using Co-IP/MS assay. STAT1 exhibited the most pronounced interaction with ESCO2 among the mass spectrometry-identified candidates. STAT1 is localized in cell cytoplasm in an inactive unphosphorylated form. Once activated, it undergoes phosphorylation and enters the nucleus, where it binds to the promoters of target genes to induce gene transcription [27].

STAT1 is essential in maintaining cell death/growth homeostasis and regulating cell differentiation under normal conditions [28, 29]. In the context of cancer, STAT1 possesses both tumor-suppressive and tumor-promotive activities [9]. In particular, the loss of activation and/or expression of STAT1 occurs in malignant cells derived from various tumors [9]. However, accumulating evidence has demonstrated the promotive effect of STAT1 in malignancies. For instance, STAT1 has been shown to facilitate the development of leukemia [30]. Moreover, dysregulated STAT1 activation\expression has been shown to assist the cancer cell immune escape and contribute to unfavorable prognosis in breast cancer [31, 32]. Additionally, our data also showed that depleting ESCO2 resulted in a downregulation in STAT1 expression (data not shown). This finding let us speculate that STAT1 may function as a downstream effector of ESCO2, which drives HPC progression by regulating, at least partially, STAT1. Furthermore, we have found that overexpressing STAT1 recovered the proliferative and migratory abilities of the ESCO2-depleted cells. Together, these findings highlighted the STAT1’s tumor-promoting role in HPC and indicated its involvement in ESCO2-mediated HPC development.

In this study, we found that ESCO2 mediates tumor progression potentially by affecting STAT1 signaling. It has been well-demonstrated that the phosphorylation-acetylation switch plays a critical role in STAT1 signaling [33]. Under this scenario, post-translational modifiers such as acetyltransferase and deacetylase are important. For instance, STAT1 acetylation has been shown to promote phosphorylation by recruiting protein kinase, which, in turn, regulates downstream signaling transduction [33]. As an acetyltransferase, ESCO2 may also contribute to regulating STAT1 signaling in HPC. We have identified a physical interaction between ESCO2 and STAT1, which suggested the crosstalk between ESCO2 and STAT1 in HPC for the first time.

Interestingly, microarray analysis demonstrated that STAT1 is co-expressed with ESCO2 and may regulate its transcription in breast cancer [23]. These findings indicate a positive regulatory loop in which STAT1 increases ESCO2 expression, further activating STAT1. However, the detailed molecular activities need further investigation.

The essential duty of ESCO2 is modifying cohesin during the S phase to stabilize the sister chromatid cohesion and gene transcription [34]. Transcription factors, like CTCF and REST/NRSF, are enriched around ESCO2 binding sites. Furthermore, the transcription of neuron-specific genes depends on the acetylation of cohesion subunits by ESCO2 [34]. In the present study, the cell cycle arrest in the S phase was apparent in ESCO2 silencing FaDu cells, which provides substantial evidence of the involvement of ESCO2 in the cell fate decision. However, our study has certain limitations as we only validated our hypothesis in one cell line. Also, the contribution of ESCO2-controlled gene transcription to the malignant progression of HPC and the specific underlying mechanisms remain enigmatic. Therefore, in future research with multiple cell lines, our focus will be to further validate and strengthen the generalizability of our results.

## Conclusion

Our study revealed an elevated ESCO2 expression in the cancer tissues of HNSC patients. The in vitro observations proved the boosting role of ESCO2 in cell proliferation, migration, and cell cycle progression and its inhibitory role in the apoptosis of FaDu cells. Moreover, ESCO2 interacts with STAT1, highlighting the potential of the JAK-STAT1 pathway being positioned downstream of ESCO2. Our data suggest that ESCO2 may serve as a new therapeutic target for HPC.

### Electronic supplementary material

Below is the link to the electronic supplementary material.

Supplementary Material 1

## Data Availability

The data and materials supporting the findings of this study are available from the corresponding authors upon request.

## Abbreviations

ESCO2: Establishment of sister chromatid cohesion N-acetyltransferase
HPC: Hypopharyngeal carcinoma
HNSC: Head and neck squamous cell carcinoma
Co-IP: Co-immunoprecipitation
MS: Mass spectrometry
STAT1: Signal transducer and activator of transcription 1
mTOR: Mammalian target of rapamycin
TCGA: The cancer genome atlas
qPCR: Quantitative real-time PCR
PI: Podium iodide
CACYBP: Calcyclin-binding protein
CUL1: Cullin 1
CDH1: Cadherin 1
HDAC2: Histone deacetylase 2
CTNNB1: Catenin beta 1
MART3: Martin 3
LGALS3: Galectin 3
CFTF: CCCTC-binding factor
REST: RE1-silencing transfection factor
NRSF: Neuron-restrictive silencer factor
