# Supplementary material for: ESCO2 promotes hypopharyngeal carcinoma progression in a STAT1-dependent manner
Source: BMC Cancer. 2023 Nov 15;23:1114. doi: 10.1186/s12885-023-11527-5 (PMC10647066; doi:10.1186/s12885-023-11527-5)
Supplement: Supplementary file 1 — Supplementary Material 1 [file 12885_2023_11527_MOESM1_ESM.docx]

**ESCO2 promotes hypopharyngeal carcinoma progression in a STAT1-dependent manner**

**Juan Hu**^1^**, Jing Yan**^1^**, Yijie Chen**^1^**, Xiaohui Li**^1^**, Liu Yang**^1^**, Haiyu Di**^1^**, Huihui Zhang**^1^**, Yewen Shi**^1^**, Junjie Zhao**^1^**, Yanxia Shi**^1^**, Yinglong Xu**^1^**, Xiaoyong Ren**^1^**, Zhenghui Wang**^1^*****

^1^Department of Otorhinolaryngology, Head and Neck Surgery, the Second Affiliated Hospital of Xi'an Jiaotong University, Xi'an (710004), Shanxi, P. R. China

***Correspondence:**

Zhenghui Wang, M.D, Ph.D

Email: [ehui4298@163.com](mailto:ehui4298@163.com)

**Figure 2B**





**Figure 5**

**CACYBP
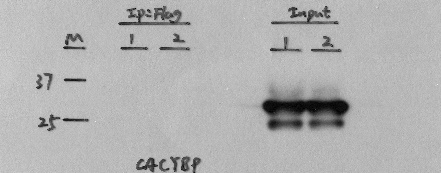
**

**CDH1
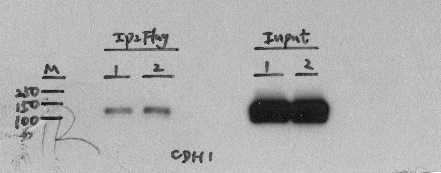
**

**CTNNB1
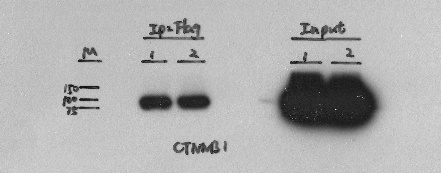

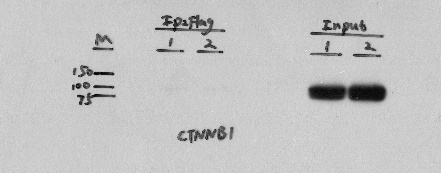
**

**CUL1
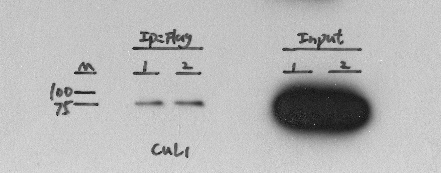

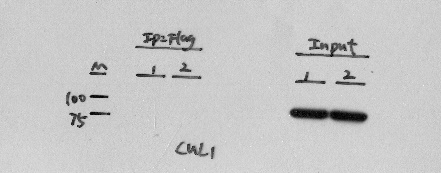
**

**EIF2B1
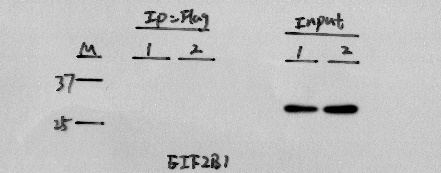
**

**HDAC2
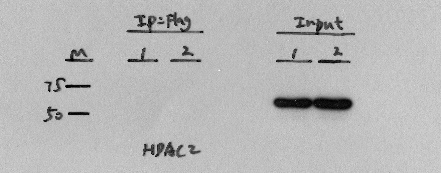
**

**LGALS3
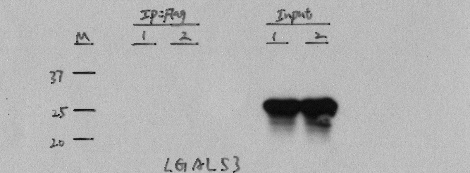
**

**MATR3
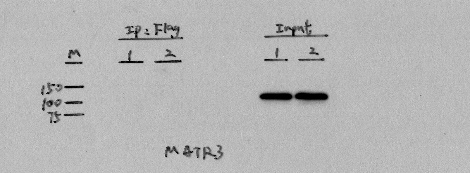
**

**STAT1
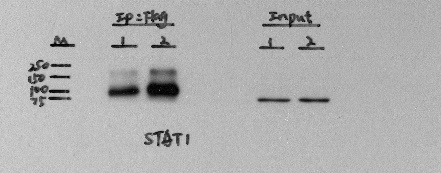
**

**Flag
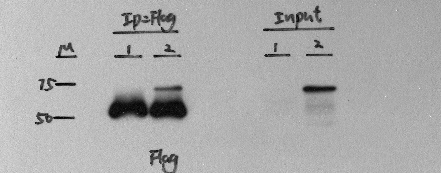
**
